# Magnetic Imaging of Encapsulated Superparamagnetic Nanoparticles by Data Fusion of Magnetic Force Microscopy and Atomic Force Microscopy Signals for Correction of Topographic Crosstalk

**DOI:** 10.3390/nano10122486

**Published:** 2020-12-11

**Authors:** Marc Fuhrmann, Anna Musyanovych, Ronald Thoelen, Sibylle von Bomhard, Hildegard Möbius

**Affiliations:** 1 Department of Computer Sciences/Micro Systems Technology, University of Applied Sciences Kaiserslautern, Amerika Str. 1, 66482 Zweibrücken, Germany; marc.fuhrmann@hs-kl.de; 2 Nanoparticle Technology Department, Fraunhofer IMM, Carl-Zeiss-Str. 18-20, 55129 Mainz, Germany; anna.musyanovych@imm.fraunhofer.de (A.M.); Sibylle.von.Bomhard@imm.fraunhofer.de (S.v.B.); 3 Institute for Materials Research, Hasselt University, Martelarenlaan 42, 3500 Hasselt, Belgium; ronald.thoelen@uhasselt.be

**Keywords:** atomic force microscopy, magnetic force microscopy, hybrid nanoparticles, polystyrene, data fusion

## Abstract

Encapsulated magnetic nanoparticles are of increasing interest for biomedical applications. However, up to now, it is still not possible to characterize their localized magnetic properties within the capsules. Magnetic Force Microscopy (MFM) has proved to be a suitable technique to image magnetic nanoparticles at ambient conditions revealing information about the spatial distribution and the magnetic properties of the nanoparticles simultaneously. However, MFM measurements on magnetic nanoparticles lead to falsifications of the magnetic MFM signal due to the topographic crosstalk. The origin of the topographic crosstalk in MFM has been proven to be capacitive coupling effects due to distance change between the substrate and tip measuring above the nanoparticle. In this paper, we present data fusion of the topography measurements of Atomic Force Microscopy (AFM) and the phase image of MFM measurements in combination with the theory of capacitive coupling in order to eliminate the topographic crosstalk in the phase image. This method offers a novel approach for the magnetic visualization of encapsulated magnetic nanoparticles.

## 1. Introduction

Magnetic nanoparticles encapsulated in a polymer matrix are of increasing importance for medical applications such as magnetic drug delivery, contrast agent for magnetic resonance imaging (MRI) and hyperthermia for cancer treatment [1,2,3,4,5]. 

Especially superparamagnetic iron oxide nanoparticles (SPIONs) are of high interest due to their unique magnetic properties. However, there is still the need for localized magnetic characterization of the encapsulated SPIONs. Magnetic Force Microscopy (MFM) has proved to be a suitable tool to image SPIONs and to map SPIONs embedded in a polymer film giving information simultaneously about spatial distribution and the magnetic behavior [6,7,8,9,10,11,12]. Passeri et al. used MFM measurements for the detection of the magnetic core of magneto ferritin and for the determination of the diameter of agglomerates in niosomes for drug delivery [13]. However, MFM measurements on nanoparticles face the difficulty that the magnetic signals interfere with topographic crosstalk because of the distance change between tip and substrate when measuring the nanoparticles [12,14]. This mirroring of surface structures in MFM phase images is still an issue in MFM research [15,16,17]. The origin of the crosstalk was experimentally proven and theoretically explained by capacitive coupling between tip and substrate [12]. This effect becomes relevant for structures smaller than the tip radius such as surface roughness or measurements on nanoparticles [18]. In interleave mode, a first scanline measures the topography of the sample, a second scanline following the topography of the first scan at a defined distance, the lift height, measures the phase image. In MFM, the phase image corresponds in principle to long range magnetic forces of the sample. However, the distance change between tip and substrate measuring above nanostructures leads to a positive phase shift indicating a positive force gradient, which might be erroneously interpreted by a repulsive magnetic force. Various methods have been suggested to minimize the topographic crosstalk. Angeloni et al. suggested a change in tip magnetization to distinguish between magnetic and electrostatic forces [19]. Analyzing the parameters relevant for the crosstalk opens several possibilities to reduce this effect [12]. Choosing a substrate with a small contact potential difference between substrate and tip, the crosstalk can be reduced. Introducing a voltage between tip and substrate to cancel the contact potential difference between tip and substrate minimizes the crosstalk as well, but the additional voltage is a further parameter and may influence the measurements [16,17]. Choosing a tip with a small tip radius also reduces the crosstalk having the disadvantage of a smaller tip magnetization. It was shown that introducing a dielectric layer between substrate and nanoparticle the topographic crosstalk can be reduced significantly because, in this case, the distance change following the topography is small compared to the overall distance between substrate and tip [18]. For measuring magnetic nanoparticles one possibility is to embed the nanoparticles in a dielectric layer in order to completely remove the topographic crosstalk [11].

In order to compensate for the topographic crosstalk in general and independently of the sample, a numerical method is needed that calculates the capacitive coupling and the topographic influence on the phase image data for each measuring point. In this paper we present the concept of data fusion of Atomic Force Microscopy (AFM) topography and MFM phase signals to correct the phase signals from topographic crosstalk. This method allows to obtain pure magnetic signals without introducing further measurement parameters such as an additional voltage and without introducing additional process steps such as the embedding of the nanoparticles. As a model system to test the concept of data fusion, unloaded polystyrene nanoparticles and polystyrene (PS) nanoparticles loaded with SPIONs are investigated. 

Data fusion on unloaded PS nanoparticles prove the concept of data fusion to compensate the topographic crosstalk completely. Data fusion on single SPIONs reveal the importance of the correction of the topographic crosstalk in order to obtain pure magnetic signals. The measurements confirm the superparamagnetic state of the SPIONs. Data fusion on PS nanoparticles loaded with SPIONs give pure magnetic signals, which show the distribution of the SPIONs in the PS capsules in accordance with Transmission Electron Measurements (TEM). It is for the first time possible to obtain spatially resolved magnetic information of encapsulated SPIONs. Only attractive forces are observed, indicating that the encapsulated SPIONs are still in the superparamagnetic state.

With the help of data fusion of AFM and MFM measurements it is now possible to discuss and interpret magnetic phase images without falsification due to the topographic crosstalk

## 2. Materials and Methods 

The synthesis of polystyrene nanoparticles is based on the method reported by Musyanovych et al. [20]. Polystyrene nanoparticles with encapsulated magnetite were synthesized through free radical miniemulsion polymerization using a ferrofluid of oleic acid-stabilized iron oxide nanoparticles (Webcraft GmbH, Gottmadingen, Germany) without further purification. In order to compare the influence of the production method on the manufactured particles, sufficient amounts of organic and aqueous phases were prepared to have identical starting conditions for the preparation of the particles. The synthesis was performed in a ratio of 1:0 and 1:0.2 polystyrene to magnetite.

For the sample preparation, the polystyrene magnetite nanoparticles in an aqueous solution were diluted with ultrapure water. Single drops of 1–3 mL of the solution were pipetted onto the substrates and allowed to dry.

MFM measurements were performed on a Bruker Dimension Icon atomic force microscope (Bruker AXS, Karslruhe, Germany). The standard methods tapping mode for topography measurements and dynamic lift mode for phase measurements were used. The lift height for measurements was 50 nm. KPFM measurements with identical tips were performed to measure the contact potential difference between substrate and measuring tip. In this work ASYMFM-HM tips were used. The collected measurement data were processed and evaluated in the SPM (scanning probe microscopy) software NanoScope Analysis (version 1.9, Bruker AXS, Karslruhe, Germany) provided by Bruker, as well as the free analysis software Gwyddion (version 2.55, www.gwyddion.net) for Data matrix extraction [21]. For data fusion and graphing, OriginPro (version 2020b, OriginLab Corp., Northampton, MA, USA) was used.

Data mapping is an important step towards eliminating topographic crosstalk and requires accurate measurement evaluation. An AFM generates a measurement image by dividing a predefined area into a discrete number of lines and scanning them one by one. The resolution of the measurement thus depends primarily on the number of lines but also on the number of measuring points per line and therefore is also size-dependent on the predefined area. Measurements were performed at a sample/line rate of 256. In this case, the scanned area is divided into 256 lines with each line having 256 measuring points. Each measurement point thus has the topography information from the first trace and the corresponding phase information from the second trace to the relevant reference point of the topography. As the measurement progresses, a data matrix is formed with X (lines) times Y (measuring points per line) measured values. In addition to the topography values, this matrix also contains phase values. Thus, for each spatially resolved point of the matrix there is topography and phase information in relation to each measuring point. It is of great importance that individual data points are exactly assigned to their measured phase. The measurement data are extracted purely numerically as a 256 × 256 data matrix. With the help of data analysis tools like OriginPro, height values can thus be linked and evaluated with those of the measured and calculated phase. The data matrix cleaned up by the topographical crosstalk can then be transferred and evaluated. A simplified form of data mapping is the linking of data within a cross-section in the area. This reduces the previously measured two-dimensional area into a one-dimensional measurement line and links the topographic measurement points of this line to the measured phase values. Using the Nanoscope Analysis measuring program, the measurement can be analyzed in two separate windows. Topography and phase values can be linked with each other by this procedure.

The morphology was investigated by transmission electron microscopy (TEM) using Zeiss Libra 120 of (Carl Zeiss NTS GmbH, Oberkochen, Germany), operating at an acceleration voltage of 120 kV. The particle dispersions were diluted with demineralized water, dropped on a 300-mesh carbon-coated copper grid and dried at ambient temperature. No additional contrasting was applied.

## 3. Theory

### 3.1. Capacitive Coupling

In interleave mode the lift height *z* between tip and surface structure is kept constant at every measuring point. Measuring small structures, for example, nanoparticles, the distance change between tip and substrate leads to a contribution to the capacity between tip and substrate depending on the topography of the structure $t_{topo}$.

The distance change between tip and substrate following the topography in the interleave mode leads in total to a positive phase shift in the MFM signal:

(1) $$\Delta \phi_{el}=-\frac{Q}{k}\left(F^{\prime }\left(z+t_{topo}\right)-F^{\prime }\left(z\right)\right)\;=\;-\frac{Q}{k}\varepsilon_{0}\left(\frac{A_{eff}}{{\left(z+t_{topo}\right)}^{3}}{\left(V_{CPD}\right)}^{2}-\frac{A_{eff}}{{\left(z\right)}^{3}}{\left(V_{CPD}\right)}^{2}\right)>0,$$

where $F^{\prime }$: force gradient acting on the tip during the MFM measurement; Q: cantilever quality factor; k: spring constant; $\varepsilon_{0}$: vacuum dielectric constant; z: lift height; $t_{topo}\left(x,y\right)$: distance parameter of topography; $t_{topo}=0$ defines the baseline of the substrate for the calculation and is the deepest point of the topography; $V_{CPD}$: contact potential difference between substrate and tip; $A_{eff}$: effective area of the capacitor responsible of the capacitive coupling.

$A_{eff}$ increases with increasing tip–substrate distance [12,14]. According to our previous work a parabolic tip shape is used to calculate $A_{eff}$ [14]. The radius of $A_{eff}$ is defined by the value of the force gradient falling below 0.1% (p=0.001) of the value of the force gradient between the top of the tip and the substrate. $A_{eff}=\pi \;r_{eff}{}^{2}$ with

(2) $$r_{eff}=\sqrt{\left(\sqrt[{3}]{\frac{{z_{1}}^{3}}{p}}-z_{1}\right)\cdot r_{tip}},$$

(3) $$z_{1}\left(x,y\right)=z+\;t_{topo}\left(x,y\right),\;$$

where *p*: percentage factor. 

Measuring the topography gives the distance parameter $t_{topo}\left(x,y\right)$ and allows the calculation of the positive phase shift due to topographic crosstalk.

### 3.2. Data Fusion of AFM and MFM

**Figure 1:**
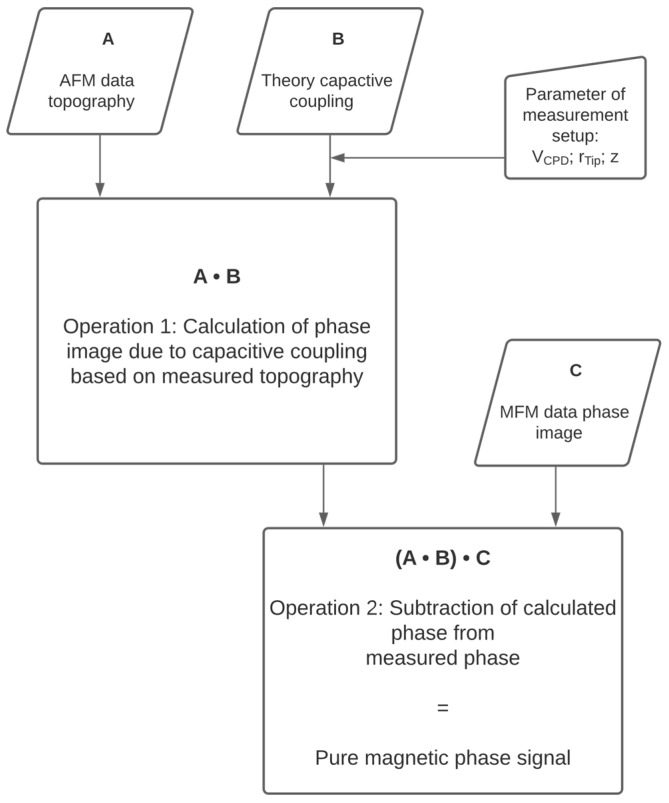
Workflow of data fusion procedure for the elimination of topographic crosstalk.

Figure 1 depicts the process of data fusion of AFM and MFM measurements for correction of topographic crosstalk in MFM phase images:

AFM topographic data are used to calculate the topographic crosstalk in MFM phase images by using the measured AFM data as $t_{topo}\left(x,y\right)$ in Equation (1) (Operation 1 in Figure 1). The exact tip radius $r_{tip}$ is determined by Scanning Electron Microscopy and $V_{CPD}$ is determined by KPFM measurements. In all measurements p=0.001 achieved the best agreement with the measured data. The calculated phase image corresponding to the topographic crosstalk is then subtracted from the measured MFM phase image data (Operation 2 in Figure 1) resulting in a phase image depicting the pure magnetic signal.

## 4. Results and Discussion

### 4.1. Measurements on Non-Magnetic PS Nanoparticles in Comparison to Measurements on SPIONs

**Figure 2:**
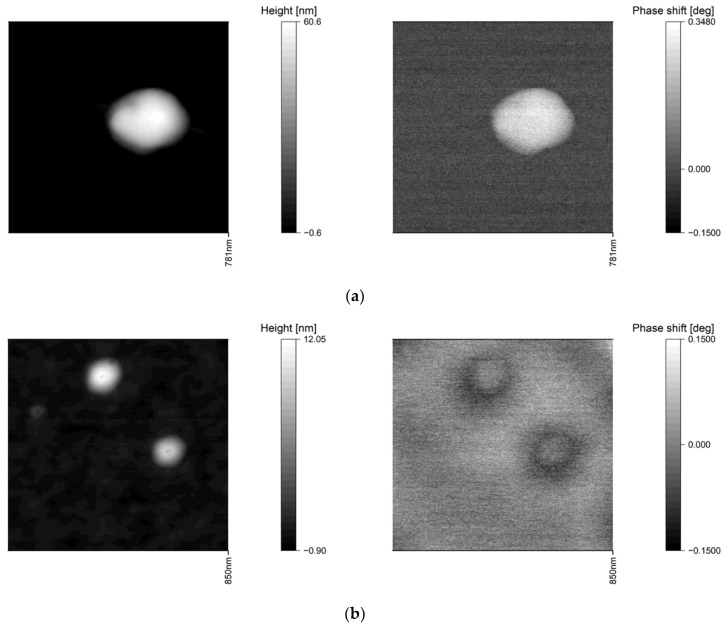
(**a**) Atomic Force Microscopy (AFM) topography (left) and the corresponding phase image in lift mode (right) of a single polystyrene (PS)-nanoparticle with a diameter of 60 nm (silicon substrate; *z* = 50 nm; *r_tip_* = 80 nm). (**b**) AFM topography (left) and the corresponding phase image in lift mode (right) of two single superparamagnetic iron oxide nanoparticles (SPIONs) with a diameter of 12 and 8 nm (silicon substrate; *z* = 50 nm; *r_tip_* = 80 nm).

All interleave mode measurements on pure PS nanoparticles as well as on single SPIONs show a topographic crosstalk in the MFM phase image. Figure 2 represents topographic AFM measurements on a single PS nanoparticle with a diameter of 60 nm and the corresponding phase image (a) and the AFM topography and phase image of two single SPIONs with a diameter of 8 and 12 nm, respectively (b):

The measurements clearly demonstrate that the interpretation of the phase image is not possible without correction of the topographic crosstalk: the non-magnetic PS nanoparticles (Figure 2a) show a positive phase instead of the expected zero phase and the SPIONs (Figure 2b) show a ring of negative phase around a positive phase instead of a completely negative phase due to their superparamagnetic character. 

**Figure 3:**
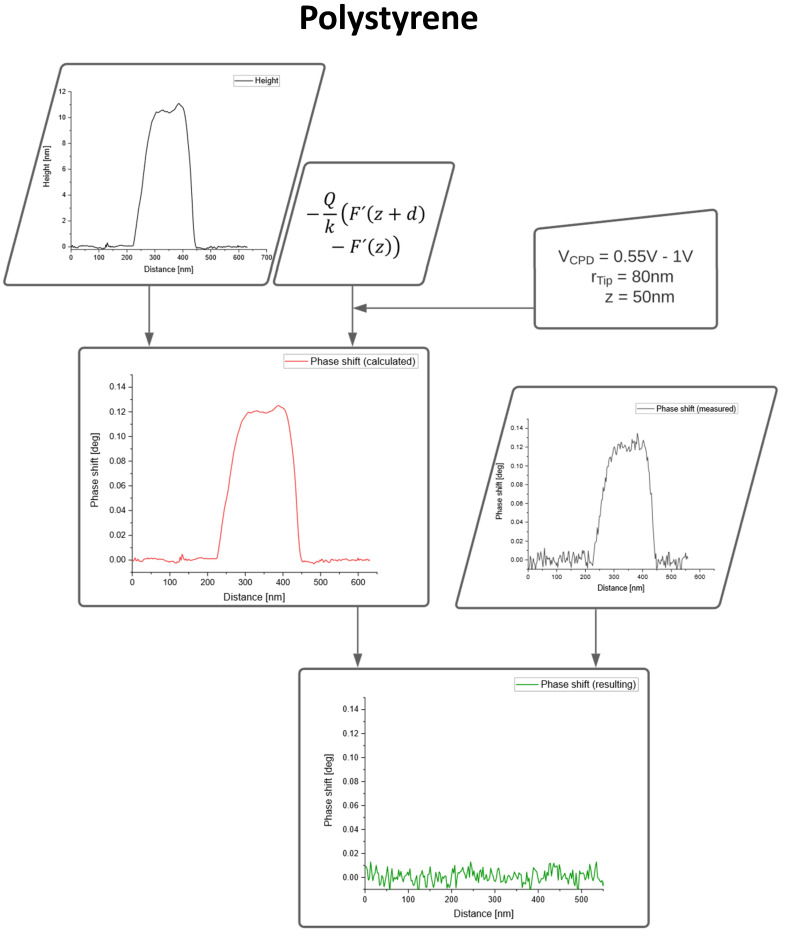
Data fusion of AFM topography and Magnetic Force Microscopy (MFM) phase image for polystyrene nanoparticles.

The data fusion process, as depicted in Figure 1, was applied to these two systems, pure PS nanoparticles and SPIONs, as shown in Figure 3. As proved in our previous work the dielectric constant of the nanoparticles has no influence on the capacitive coupling and, therefore, has not been taken into account [14]. The roughness of the substrate (on average around 1 Å) is small compared to the size of the nanoparticles and therefore does not contribute significantly to the topographic crosstalk.

For pure PS particles the topographic crosstalk is compensated completely, resulting in a phase image of zero as expected for non-magnetic nanoparticles, as shown in Figure 3.

**Figure 4:**
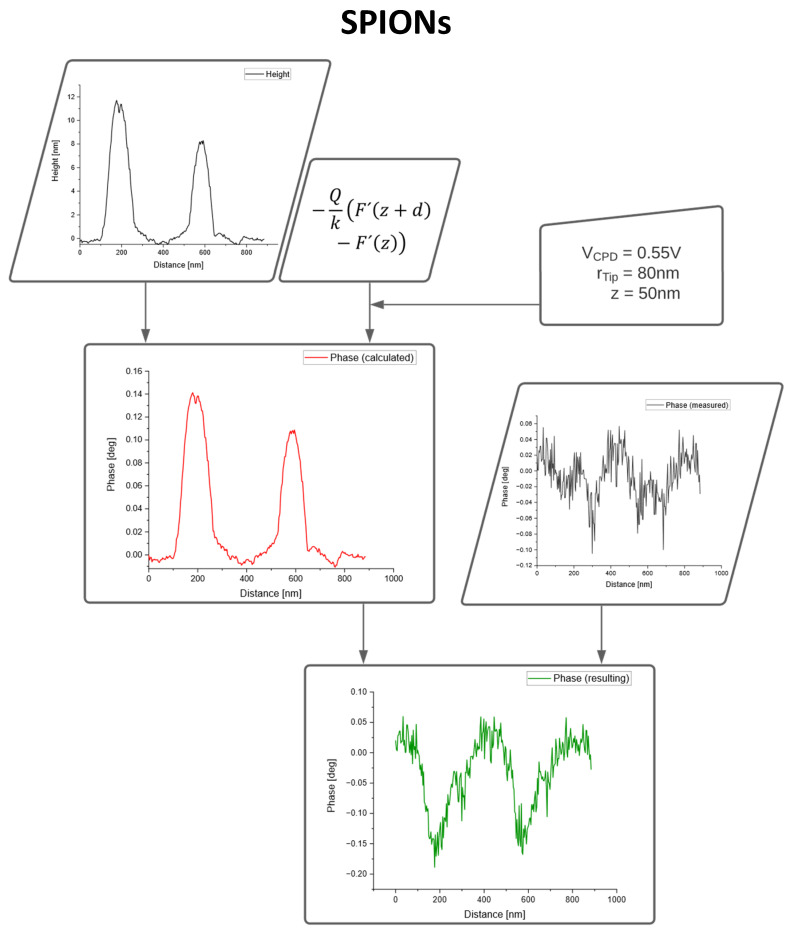
Data fusion of AFM topography and MFM phase image for SPIONs.

The charts in Figure 4 illustrate the correction of the topographic crosstalk for SPIONs.

**Figure 5:**
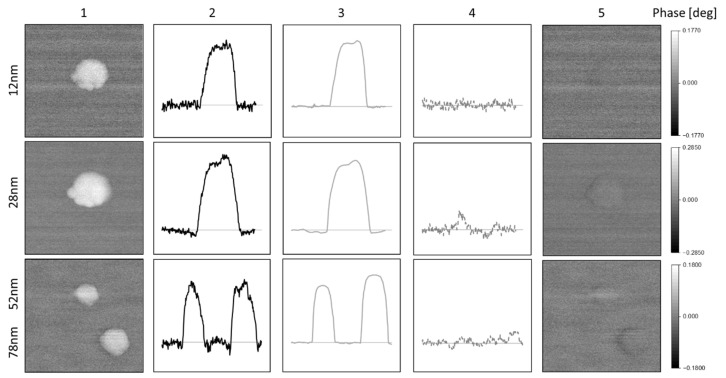
Data Fusion of different polystyrene capsules (top to bottom): (**1**) measured phase shift, (**2**) cross-section of measured phase shift, (**3**) calculated phase shift based on topographic cross-section, (**4**) cross-section of resulting phase shift by data fusion, (**5**) resulting phase image. (Silicon substrate; *V_CPD_* = 0.55 V − 1 V; *z* = 50 nm; *r_tip_* = 80 nm).

The magnetic phase image only shows negative values as expected for superparamagnetic particles. Figure 5 demonstrates the process of data fusion for PS particles with different diameters ranging from 12 to 78 nm.

As the polystyrene nanoparticles are non-magnetic, the measured positive MFM phase image clearly indicates significant contributions due to capacitive coupling (column 1). The second column shows cross-sections through the phase images of the measured data. The third column contains the calculated phase shifts (Operation 1 in Figure 1) due to capacitive coupling and based on the topography AFM measurements. The fourth column presents the results of Operation 2, the subtraction of measured and calculated phase in Figure 1, in form of a cross-section. It is clearly seen that the topographic crosstalk is eliminated and the phase signal approaches the measurement noise. The fifth segment visualizes the elimination of the crosstalk in the two-dimensional phase images. For all particles, the topographic crosstalk is almost completely removed. 

These measurements prove that the process of data fusion is an appropriate method for elimination of topographic crosstalk for a wide range of nanoparticle sizes.

### 4.2. Measurements on SPIONs Encapsulated in PS

**Figure 6:**
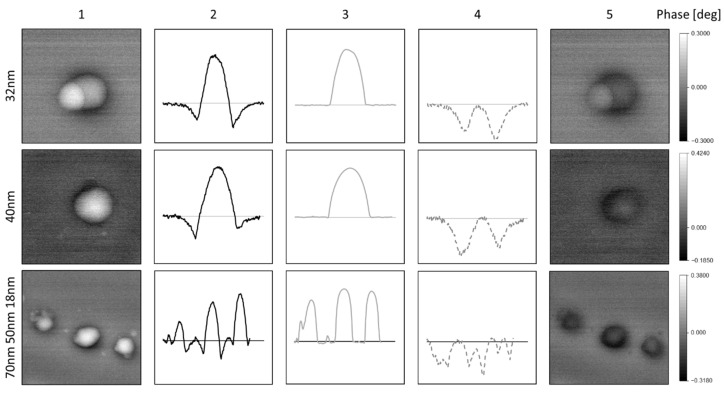
Data fusion for polystyrene particles filled with magnetite (ratio 1:0.2) with different diameters (top to bottom): (**1**) measured phase shift, (**2**) cross-section of measured phase shift, (**3**) calculated phase shift based on topographic cross-section, (**4**) cross-section of resulting phase shift by data fusion, (**5**) resulting phase image. (Silicon substrate; *V_CPD_* = 0.55 V; *z* = 50 nm; *r_tip_* = 80 nm).

Data fusion of AFM and MFM measurements now allow to investigate the localized magnetic behavior of encapsulated SPIONs. SPIONs with a diameter ranging from 7 to 10 nm diameter are encapsulated in PS nanoparticles with a diameter from 18 to 100 nm. The measured phase images (column 1) show a positive phase shift (white color) surrounded by a ring of negative phase shift (black color). The calculation of the topographic crosstalk based on the topographic AFM-measurements demonstrates that the measured positive phase shift is not due to repulsive magnetic forces but only due to topographic crosstalk (column 3 in Figure 6). Removing the topographic crosstalk, the corrected phase images only show negative values indicating the superparamagnetic character of the SPIONS.

**Figure 7:**
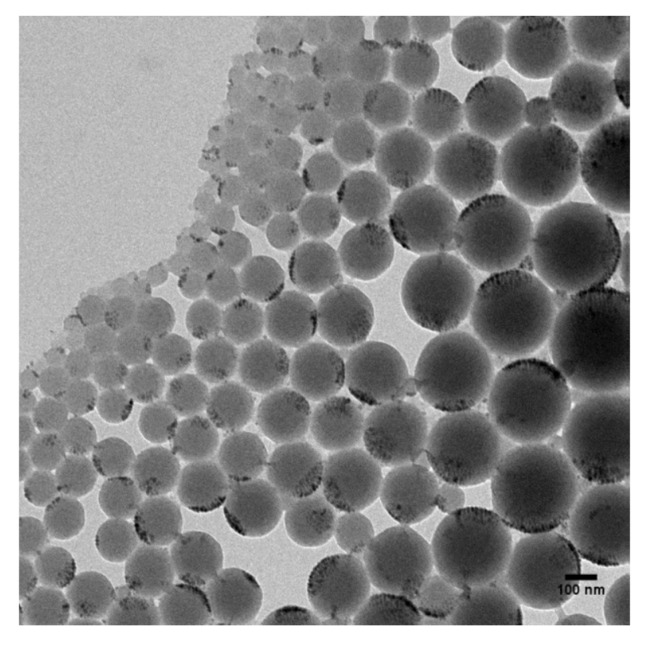
Transmission Electron Measurements (TEM) image of PS nanoparticles with encapsulated SPIONs.

The corrected phase images of PS nanoparticles show that the SPIONs are located at the outer edge of the PS nanoparticles, which is in good agreement with TEM measurements shown in Figure 7. The pure magnetic signals (columns 4 and 5 in Figure 6) only show attractive forces indicating that the encapsulated SPIONS are still in their superparamagnetic state in accordance to VSM measurements on similar nanoparticles [21].

## 5. Conclusions

In summary, a numerical method was developed, which in general allows the correction of topographic crosstalk in MFM measurements. This method is based on data fusion of the AFM topography and the MFM phase image in combination with the theory of capacitive coupling. 

The success of the data fusion was demonstrated by measurements on pure polystyrene nanoparticles of different sizes serving as a non-magnetic model system. With this method it was then possible to magnetically characterize in SPIONs encapsulated in polystyrene. The measurements demonstrate the superparamagnetic behavior of the SPIONs. It is now possible to magnetically image encapsulated SPIONs without falsifications due to the topographic crosstalk. The correction by data fusion presented in this paper thus offers a solution not only for nanoparticles but also for various applications that are affected by topographic crosstalk in lift mode measurements. An implementation of the measurement software is conceivable and could be directly incorporated into the analysis by an automated background process.
